# Approach to Mental Health Through a Frequency Modulated Auditory Intervention: A Controlled and Randomized Clinical Trial

**DOI:** 10.3390/jcm14010288

**Published:** 2025-01-06

**Authors:** Beatriz Estalayo-Gutiérrez, María José Álvarez Pasquín, Francisco Germain

**Affiliations:** 1 Servicio Madrileño de Salud, José María Llanos University Health Centre, 28053 Madrid, Spain; 2 Servicio Madrileño de Salud, Santa Hortensia University Health Cetre, 28002 Madrid, Spain; malvarezp@salud.madrid.org; 3 Department of Systems Biology, Alcalá de Henares University, 28871 Madrid, Spain

**Keywords:** auditory perception, anxiety-depression, emotional well-being, auditory intervention, Bérard method

## Abstract

**Objective**: The clinical trial Effect of Modulated Auditory Stimulation on Interaural Auditory Perception (NCT0544189) aimed to determine whether an auditory intervention (AI)—“Bérard in 10”—can enhance the effect of standard therapies for people with anxiety and/or depression. **Methods**: Design: unblinded, randomized, controlled clinical trial. Location: Mejorada del Campo Health Centre, Madrid (Primary Care). Participants: A total of 233 patients selected by systematic sampling and meeting the following selection criteria: age of majority, absence of severe acute pathology or chronic decompensated pathology. They were evaluated with the Goldberg and Hamilton tests and classified into the Emotional Well-Being group (EWB, *n* = 86) or the Anxiety and/or Depression group (AD, *n* = 147). Just half of each group received an AI. Intervention: Listening to classical music processed through a frequency modulator (Earducator) to attenuate abnormal frequencies, 30 min per session, two sessions a day for 5 days. Main measurements: Hamilton Tests for Anxiety and Hamilton Test for Depression, at 3 and 6 months. **Results**: In the analysis by protocol, EWB with AI (*n* = 14) obtained lower scores in anxiety and depression at 3 and 6 months than EWB without AI (*n* = 36) (*p* < 0.05), the effects being large and moderate, respectively; AD with AI (*n* = 31) had lower scores on anxiety and depression at 3 months and anxiety at 6 months than AD without AI (*n* = 52) (*p* < 0.05), the effect being small. No damage reported. **Conclusions**: The AI “Bérard in 10” significantly prevents the onset of anxiety and depression and somewhat improves the effect of standard treatments in primary care.

## 1. Introduction

Mental health disorders are a major global problem due to the economic, social and personal costs they generate [1]. About 10% of the world’s population is affected. In addition, there has been an increase of almost 50% in recent years [2], the COVID-19 pandemic having a devastating effect in this regard [3].

The prevalence of mental pathology in Europe varies among countries and over time [1,4], as well as people’s access to a primary care network [5,6]. In Spain, around 49.2% of the population attending primary care have mental disorders [7]. This level of care seems the logical place to respond to most mental health problems, due to its accessibility, longitudinal doctor–patient relationships, biopsychosocial vision and capacity to coordinate additional services beyond its field of experience and knowledge [2].

Within mental health disorders, anxiety disorders and depression are the most frequent entities [8]. There is a significant comorbidity of both in primary care, and it remains unknown whether this comorbidity is due to two different pathological processes or a single one [9].

The treatment of anxiety can be psychological and/or pharmacological [10]. The first is fundamentally based on cognitive-behavioral therapy (CBT). The second includes selective serotonin reuptake inhibitors, noradrenaline inhibitors and mirtazapine. The combination of both approaches seems to be the most effective alternative [10]. In addition, there are non-pharmacological adjuvant treatments with different degrees of evidence, whose application is recommended in the absence of side effects: relaxation techniques, mindfulness, yoga, aromatherapy, therapy through images and use of new technologies, among others [11]. Their results are modest [12,13,14,15,16].

The treatment of depression encompasses psychotherapeutic, psychosocial and pharmacological interventions [17,18]. CBT is the fundamental psychological approach, while pharmacological treatment includes mainly selective inhibitors of serotonin, monoamine oxidase, norepinephrine and dopamine reuptake. The combination of cognitive behavioral therapies with antidepressants achieves better results [17,18]. Non-pharmacological treatments of depression like naturopathic therapy, biological interventions and physical activity interventions seem to help reduce the symptoms of depression in combination with the classic approach. Nevertheless, scientific evidence on this is scarce [19].

Lately, music has been used in the treatment of mental illnesses such as schizophrenia, bipolar disorder, generalized anxiety disorder, major depressive disorder or post-traumatic stress disorder [20]. In this sense, it seems that music reduces anxiety-depressive symptoms, possibly because it affects neural circuits such as reward circuits, arousal, emotional regulation, learning and functional neuroplasticity [21]. In addition, its beneficial effect has been observed in healthy people by promoting interbrain connections [15]. On the other hand, a correlation has been observed between the type of emotional alteration and the modification of the normal auditory pattern. Thus, while in anxiety there is an increase in hearing thresholds for low frequencies and a decrease for high frequencies [22,23], in depression, an elevation of hearing thresholds has been found in the highest frequencies [23,24]. All of this means that the interaural difference is greater in anxious-depressive disorders than in emotional well-being. These changes depend on the ear in question. Thus, in anxiety, hearing loss is observed mainly in the right ear, while in depression, hearing impairment affects both ears [23]. An auditory intervention (AI) could modify the interaural asymmetry (different hearing thresholds when comparing the right and left ear) as well as the auditory thresholds of people [25]. AI smoothed out high-frequency prioritization and seemed to balance hearing between ears. However, it remains unknown whether these changes had any effect on anxiety and/or depression disorders.

The aim of this research is to determine if this AI can enhance the effect of other therapies followed by patients with anxiety and/or depression in a health center. This therapy was administered in a complementary way to other pharmacological and psychological treatments, since for ethical reasons they could not be suppressed. This experimental design allowed us to verify whether it acts by a different mechanism than conventional therapies. Likewise, demographic and clinical factors associated with a better therapeutic response were analyzed.

## 2. Materials and Methods

### 2.1. Study Type

This study is an unblinded, randomized, controlled clinical trial, registered as Effect of Modulated Auditory Stimulation on Interaural Auditory Perception, number NCT05441891. The study compared an auditory intervention versus non-intervention in people with emotional well-being and individuals experiencing symptoms of anxiety and/or depression [25].

The protocol received approval from the Ethics Committee of Hospital la Princesa, Madrid, on 8 March 2012 (project identification code: 05/11), and subsequently by the Local Research Committee of Southeast Madrid [25].

The criteria for good practice for clinical trials were followed at all times, with informed consent being requested prior to inclusion and observation of the Declaration of Helsinki’s guidelines in its latest version.

### 2.2. Sample Size Determination

A sample of 124 was determined, with half allocated to the emotional well-being group and the other half to the anxiety and/or depression group, after considering a bilateral test, with a confidence level of 95%, a statistical power of 80% and an expected proportion of losses of 15%.

### 2.3. Population

A total of 327 individuals were selected from the lists of people who requested an appointment at the Centro de Salud Mejorada del Campo (Madrid, Spain). This selection was carried out through systematic sampling. Basically, the first of every five people from said lists who attended 6 different family medicine consultations were chosen, with a distribution of 3 people in the morning and another 3 in the afternoon. The inclusion criteria were subjects of legal age who signed informed consent. The exclusion criteria included pregnancy, deafness, serious or psychotic illness, as well as drug or alcohol consumption [23]. Compliance with the selection criteria was verified through a personal interview conducted by a family doctor.

Prior to any test, subjects’ ears were examined and any earwax plugs removed. Later, the Goldberg Scale was passed as a screening tool for anxiety and depression symptoms. Those without significant anxiety and depression scores on the GADS were included in the “Emotional Well-Being” group (EWB), while those with significant anxiety and/or depression scores went on to complete Hamilton Rating Scale questionnaires and were classified in the Anxiety and/or Depression group (AD). Finally, each participant filled out a questionnaire on sociodemographic and clinical data [23].

### 2.4. Assignment to Study Groups

Each emotional state group was divided into an intervention subgroup and a non-intervention subgroup by means of a stratified block randomization [25].

No one involved in the randomization process was in charge of applying the treatments, collecting the evaluations or analyzing the study data. Only those subjects who met the selection criteria and signed informed consent were randomized.

### 2.5. Auditory Intervention (AI)

The auditory intervention consisted of an adaptation of the Bérard method [26], reducing the number of sessions from 20 to 10 (“Bérard in ten”): 10 sessions of 30 min listening to music modulated by a frequency equalizer (Earducator) divided into two daily sessions separated by at least 3 h. The technical details of the intervention have been previously published [25].

### 2.6. Absence of Hearing Intervention

No intervention was applied to participants without an intervention assignment.

### 2.7. Study Variables

The Goldberg Anxiety and Depression Scale was used as a screening instrument in the evaluation of emotional disorders [27], while to evaluate the intensity and frequency of the disorders, the Hamilton Scale for Anxiety (HAS) and the Hamilton Scale for Depression (HAMD) were applied [28]. The Spanish validated versions were used for this purpose [29,30]. The patient’s condition was evaluated in the previous 3 weeks.

#### 2.7.1. Hamilton Anxiety Scale—Total Anxiety

This score corresponds to the 14 items of the Hamilton Anxiety Scale. The range goes from 0 to 56 points.

#### 2.7.2. Hamilton Anxiety Scale—Psychological Anxiety

Items 1–6 of the Hamilton Anxiety Scale were scored. Range: 0 to 24 points.

#### 2.7.3. Hamilton Anxiety Scale—Somatic Anxiety

Items 7–14 of the Hamilton Anxiety Scale were scored. Range: 0 to 32 points.

#### 2.7.4. Hamilton Depression Scale

The score of 17 items of the Hamilton Depression Scale was obtained. Range: 0 to 52 points.

#### 2.7.5. Sociodemographic Variables

Data on nationality, gender, age, educational background, work status, sound exposure and safeguarding and medical history were collected from the individual participants.

Sociodemographic variables were collected at baseline, while Hamilton’s Anxiety and Depression Scales were applied at baseline, at 3 months and at 6 months to each participant.

### 2.8. Response Quantification

The total response to treatment was defined as a ≥50% decrease in the initial scale score and partial response as a decrease between 25–49%, while non-response as a reduction of <25%.

### 2.9. Statistical Analysis

The data analysis was conducted exclusively on subjects who successfully completed the entire process and intervention. The distribution was verified to be normal using the Shapiro–Wilks test, and homoscedasticity was studied using the Levene test, which was not significant. The means were statistically compared using two-way ANOVA and the Bonferroni post hoc test.

Contingency tables (Fisher’s exact test) were used to analyze the association between the variables due to auditory intervention and the different previous circumstances (taking analgesics, etc.). Statistical significance was considered *p* < 0.05.

Data analysis was carried out with GraphPad Prism for Windows version 9.0.0 (GraphPad Software, San Diego, CA, USA).

## 3. Results

**Figure 1:**
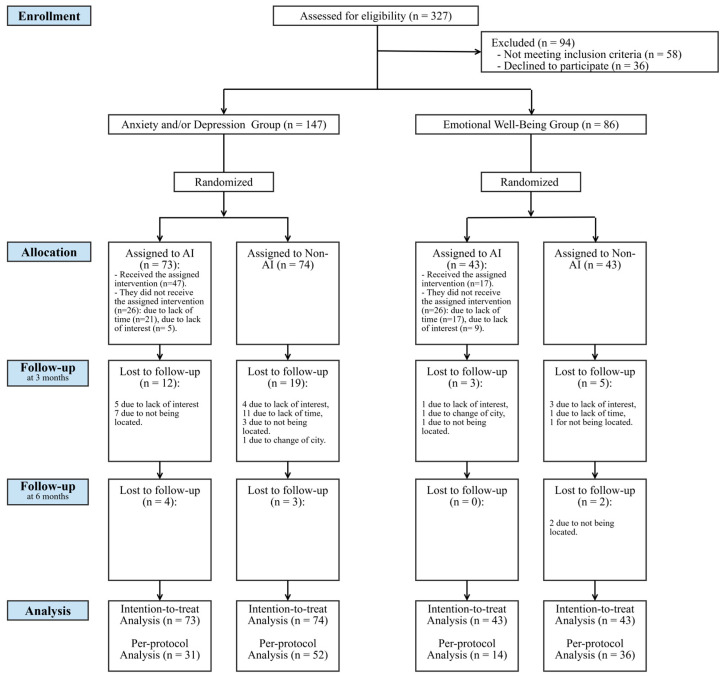
Flowchart of the clinical trial. An unblinded, randomized, controlled clinical trial. AI: Auditory Intervention.

The flow of participants throughout the study is shown in Figure 1.

Since psychological conditions can naturally evolve into other states, the extent to which such changes occurred in the study groups at three and six months after the start of the study was analyzed.

Part of the Emotional Well-being group without AI, three months after starting the study, experienced a progression towards Anxiety and/or Depression (37%). At six months, the number of people with Anxiety and/or Depression increased (45.7%). The group with Anxiety and/or Depression, both three and six months after the start of the study, experienced an evolution towards Emotional Well-being in a small percentage (6.8%). It is noteworthy that the number of patients with depression alone was minimal.

The number of intervention participants who completed the 10 auditory intervention sessions was 14 in the Emotional Well-being group and 31 in the Anxiety and/or Depression group.

### 3.1. Demographic and Health Characteristics According to the Intervention Group

The characteristics of the groups are displayed in Table 1.

After analyzing the possible associations between the application of AI and the previous circumstances (taking analgesics, immunological, anxiolytic, antidepressant, psychological treatment and liking for music), only liking for music showed a significant response in the A/D group (*p* = 0.0113).

### 3.2. Internal Consistency of the Psychometric Scales

The analysis carried out determined that the internal consistency reliability coefficient (Cronbach’s alpha) value was 0.87 for the Hamilton Total Anxiety Scale, 0.86 for the Psychological Anxiety Scale, 0.84 for the Somatic Anxiety Scale and 0.79 for the Hamilton Depression Scale. These results show good internal consistency for the questionnaires.

### 3.3. Effect of Hearing Intervention on Anxiety and Depression in the Two Emotional Groups

Since these variables followed a normal distribution, repeated measures two-way ANOVA with a Bonferroni post-test were used for analysis.

To determine the preventive capacity that AI may have on the development of anxiety or depression states, the different variables of the Anxiety and Depression tests in the Emotional Well-being group were compared over time between the subgroup that received the intervention and the subgroup that did not receive it. To determine the therapeutic capacity of AI, the same comparison was carried out in the Anxiety and/or Depression group between the subgroup that received the intervention with that which did not receive it. The compared psychological variables were measured at the initial moment (T0), at 3 months (T3m) and at 6 months (T6m).

#### 3.3.1. Comparison of Means at Different Time Milestones

The means of the HAS-Total, HAS-Psychological, HAS-Somatic and HAMD variables were compared at baseline and at 3 or 6 months for each of the intervention subgroups of the EWB and AD groups.

**Figure 2:**
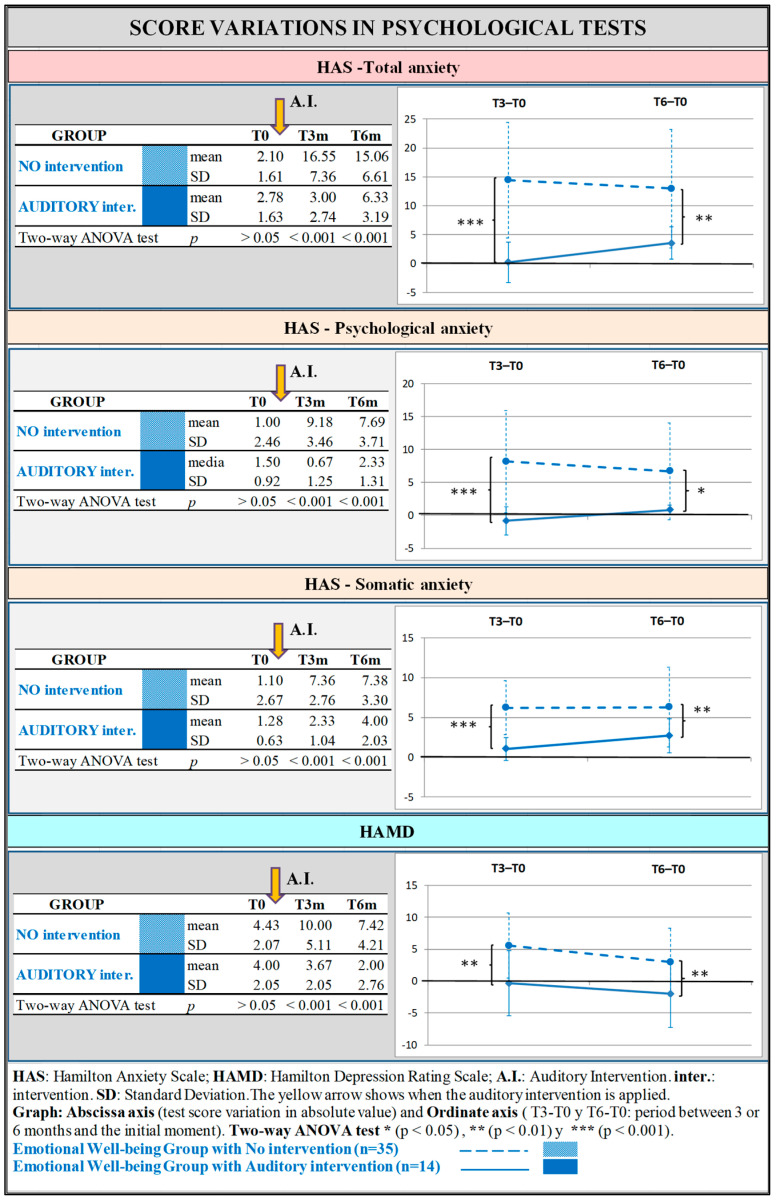
Emotional Well-being group: Evaluation of anxiety and depression in the subgroups with and without Auditory Intervention using Hamilton Test for Anxiety and Depression. The tables in the left column correspond to the absolute values obtained in the psychological tests. The figures in the right column compare for the two groups (the one in which the AI was applied and the one in which it was not applied) the difference between time 3 months and time 0, and that between time 6 months and time 0.

The EWB group showed statistically significant results for all the scales, both at 3 and 6 months. The proportion of change was more important at 3 months. The subgroup that achieved a lower score (indicating a reduced risk of anxiety and depression) was always the one that had received the AI (Figure 2).

**Figure 3:**
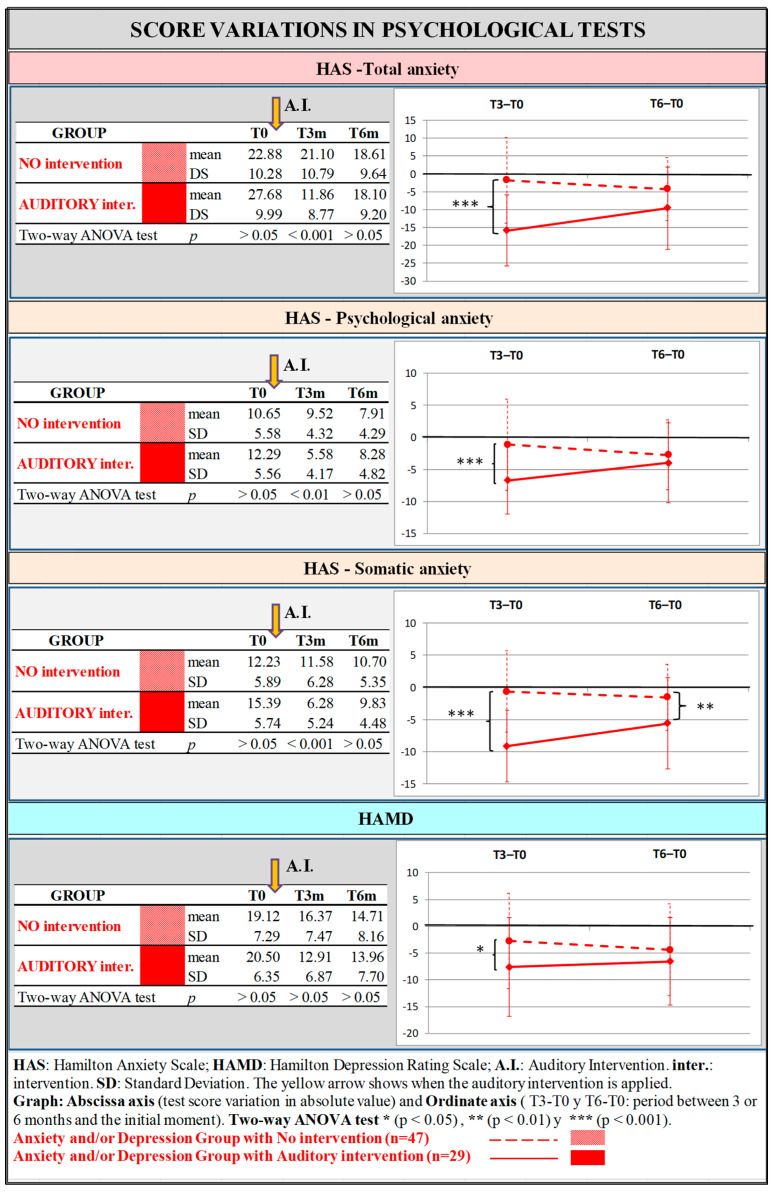
Anxiety and/or Depression group: Evaluation of anxiety and depression in the subgroups with and without Auditory Intervention using Hamilton Test for Anxiety and Depression. The tables in the left column correspond to the absolute values obtained in the psychological tests. The figures in the right column compare for the two groups (the one in which the AI was applied and the one in which it was not applied) the difference between time 3 months and time 0, and that between time 6 months and time 0.

In the AD group, all studied scales showed statistically significant differences 3 months post-intervention; however, 6 months after the intervention, the differences were significant only for HAS-Som. The AD subgroup with the best results was the one that had received the AI (Figure 3).

#### 3.3.2. Response to Treatment

Table 2 presents the study groups’ response levels to the Hearing Intervention.

#### 3.3.3. Standardized Mean Difference

To know the size of the effect and make it comparable with other interventions, Cohen’s d or Standardized Mean Difference was calculated (Table 3). The combined standard deviation was used for groups of different sizes. The sizes of the effect, small, medium and large, are indicated by standardized mean differences of 0.2, 0.5 and 0.8, respectively.

Auditory intervention with 10 sessions showed a great effect in preventing anxiety at 3 months, but only moderate at 6. It was also moderate in preventing depression at 3 and 6 months (Table 3). From a curative point of view, AI had a small effect in reducing anxiety in the AD group at 3 months (Table 3).

#### 3.3.4. Epidemiological Factors Associated with a Better Response After Auditory Intervention

The effect that the different epidemiological factors had on the different groups studied after the hearing intervention can be seen in Table 4.

In patients without hearing intervention, the factors associated with a better evolution were those with an age between 18 and 51 years, university studies, not retired, without a family history of depression, chronic pain or mental pathology and without consumption of analgesics, antidepressants nor follow-up of psychological treatment in the past. These results were common at both 3 and 6 months. The patients with hearing intervention who presented the best results were those in the fourth and fifth decades of life, more than primary studies, jobless, no history of mental pathology and not having taken anxiolytics or antidepressants in the past, nor currently taking painkillers.

#### 3.3.5. Adverse Effects

No adverse effects were recorded during or after hearing intervention.

## 4. Discussion

The auditory intervention prevented symptoms of anxiety and depression at both 3 and 6 months in the EWB group. On the other hand, in the AD group, at 3 months, these symptoms were reduced, while at 6 months only somatic anxiety decreased.

In the groups without hearing intervention, a certain tendency to change was also observed. Although this trend was much greater in the Emotional Well-being group, this is logical if we consider that this sample comes from patients who go to a health center for some health problem.

Different musical techniques have been used to treat anxiety and depression [20,31,32], but they have only shown a small effect with a moderate degree of evidence. In our case, the AD group also showed a small effect on the control of anxiety and depression. This is probably due, at least in part, to the fact that they are a more heterogeneous group, decreasing the power of study. Another compelling reason may be the intensity and duration of symptoms, which were not controlled in this study. Previous scientific evidence shows that musical interventions improve depressive symptoms and anxiety somewhat less [20]. This evidence was obtained from the hospital setting. In our primary care-based study, the effect detected seems to be the other way around, with a greater reduction in anxious symptoms than depressive symptoms. This is probably due to a higher proportion of anxious symptoms than depressive symptoms in the AD sample, which reflects the reality of those attended in health centers [8,9].

Our study shows that the hearing intervention “Bérard in ten” is more preventive than curative, which is consistent with previous studies [33]. In the present study, the preventive effect of a musical intervention on the symptoms of anxiety and depression has been observed in a novel way, which is even greater than the preventive effect demonstrated by physical exercise in a healthy young population [13].

The conduct of the study and its possible applications in the field of primary care are also novel. The total absence of reported side effects is interesting. On the other hand, the high dropout rate is like that obtained in acute treatments (such as antibiotics) or chronic treatments (such as antihypertensives and antidiabetics) in primary care [34]. The proportion of losses was higher (29.6%) than expected (15%), possibly due to the long duration of the study, 6 months, compared to the 3 months that had been carried out in previous studies [26]. The EWB presented a lower proportion of losses (19.77%) than the AD group (35.37%), possibly due to the patients’ need for immediate results, and with the least possible effort with symptoms of anxiety and/or depression.

There are several reasons to include patients with symptoms of anxiety and/or depression in the same group. Firstly, both share functional alterations in the locus coeruleus, the raphe nuclei and the limbic system. These alterations tend to produce confusion and distortion of perception through the amygdala, especially auditory and visual perceptions [35]. Furthermore, epidemiologically, there is a frequent comorbidity between anxiety and depression [36,37]. On the other hand, in primary care, the patient’s suffering is addressed through a biopsychosocial action plan rather than from a precise diagnosis [38,39,40].

The analysis of the epidemiological factors of the Anxiety and/or Depression group showed a greater proportion of women with an average age of 45 years, primary education and in a working situation, which was similar to that of other studies [41,42]. It also has a higher proportion of retirees and a greater active consumption of painkillers. Other important factors are pain [42] and early retirement as possible causes in the development of depression.

The hearing intervention obtained better results in the unemployed population, which is encouraging, since there is a strong association between unemployment and anxiety-depression [43,44]. Among the possible causes of this therapeutic effect could be having a greater availability of time to follow the therapy. Additionally, the lack of prior consumption of anxiolytics and/or antidepressants favors the control of anxiety symptoms by the intervention. This fact could be due to the absence of severity and/or chronicity of the symptoms or neurological traces of past processes, as suggested by the previous consumption of these medications. Furthermore, the control of depressive symptoms at 6 months improved if there was no current mental pathology, since said mental pathology is a source of significant anxiety and depression [36,45]. On the contrary, the current consumption of analgesics worsened anxiety control after the intervention, possibly because the pain and/or the analgesic itself could modulate the action of the auditory intervention on hypothetical brain connections or auditory mechanisms. In the same way, the negative effect of anti-inflammatories on hearing [46] and chronic pain on the central nervous system [47] are known. Regarding age, there is better control of anxiety in the population aged 31 to 50 years compared to those aged 51 to 65 years, which could be due to less chronicity of anxiety symptoms. Finally, having completed university studies predisposes one to a better socioeconomic position and, secondarily, to a lower prevalence of anxiety disorders [48].

All other factors (family and personal history) would favor early symptoms of anxiety and/or depression and, consequently, permanent functional changes in the neurological circuits that govern them. These findings are similar to those of previous studies [49,50].

The Bérard method was known in the 1980s, and its application focused on autism and in the school environment to help children with learning difficulties [51]. Although initially, the studies with positive results were numerous, subsequent systematic reviews did not demonstrate sufficient scientific evidence [52,53]. The main methodological limitations were lack of statistical power and lack of control groups. Due to this fact, its use has been progressively declining. As for mental health, only a series of cases have been reported to treat depression, although with promising results [51]. In this context and being aware of the scientific evidence between music and mood, we wanted to evaluate the Bérard method in the control of anxiety-depressive symptoms. For this purpose, we considered a control group essential, as well as a sufficiently large sample that would allow good statistical power. The losses during the study somewhat diminished this last objective.

Many theories have attempted to explain the effects of auditory interventions such as those of the Bérard method [26]. They focus on different areas of the auditory pathway. Listening to music is known to activate complex neural networks beyond the auditory cortex, including vast temporal, frontal, parietal, subcortical and cerebellar areas [15], but it is not known how auditory intervention can modulate these neural networks. We postulate that the auditory intervention “Bérard in ten” reverses the prioritization of high frequencies perception in the right hemisphere through exposure to intense stimuli of unexpected and alternating presentation, exhausting the state of audiological hyperalertness [25].

The strengths of this study lie in its design as a randomized controlled clinical trial, allowing the study not only of the curative effect but also of the preventive effect of auditory intervention; longitudinal follow-up of patients up to 6 months, when to date, only a maximum period of 3 months has been evaluated; and the location of the study in the field of primary care, where the prevalence of anxiety and depression is high and its accessibility allows frequent approaches. Regarding the weaknesses of the study, we point out that the design is open (not blinded), which prevents discerning the placebo effect of the intervention; the heterogeneity of the AD group in which patients with symptoms of anxiety only, depression only and symptoms of anxiety plus depression are grouped. In addition, it is not controlled if the symptoms of anxiety and depression are acute, chronic or in remission. Finally, the concomitance of other pharmacological or psychotherapeutic interventions is a weakness, which prevents the evaluation of the net effect of the auditory intervention.

As for future directions, the auditory intervention practiced is based on the Bérard method, which was carried out in 20 sessions. It is possible that doubling the sessions received by the participants and with a sufficiently large sample size would achieve more significant and lasting results. It would be interesting to explore modifications of the “Bérard in ten” AI that would allow self-application by the patient at home. In addition, the incorporation of tools for measuring attitudes and musical skills in individuals would allow the effect of music on anxiety and depression levels to be analyzed in a more personalized way. Finally, it would be interesting to explore the effect of the “Bérard in ten” method on memory or habituation, even combining it in a regulated way with another therapeutic approach (such as CBT) by studying its effect on anxiety-depressive symptoms in the short and medium term.

## 5. Conclusions

The auditory intervention is able to prevent anxiety and/or depression in people with emotional well-being, as well as to have a certain palliative effect on people in the anxiety and/or depression group. Thus, the auditory intervention (“Bérard in ten”) is an effective, brief and harmless preventive tool to take into account in the management of anxiety and/or depression, especially in the primary care setting.

## Figures and Tables

**Table 1:** Demographic and health characteristics by intervention groups.

	Anxiety and/or Depression Group (*n* = 83)		Emotional Well-Being Group (*n* = 50)	
	Auditory Interv. (*n* = 31)	Non-Intervention (*n* = 52)	Auditory Interv. (*n* = 14)	Non-Intervention (*n* = 36)
Age (years old)	50.40 (11.30)	42.49 (12.02)	40.33 (22.50)	46 (12.91)
Gender (female)	23 (74.19)	39 (75)	5 (35.71)	17(47.22)
Nationality				
Spanish	29 (93.54)	45 (86.53)	14 (100)	33 (91.66)
Rest of Europe	1 (3.22)	4 (7.69)	0 (0)	3 (8.33)
African	0 (0)	0 (0)	0 (0)	0 (0)
Hispanic American	1 (3.22)	3 (5.76)	0 (0)	0 (0)
Education Level				
Without	0 (0)	3 (5.76)	1 (7.14)	0 (0)
Primary studies	6 (19.35)	27 (51.92)	10 (71.43)	16 (44.44)
Secondary studies	0 (0)	12 (23.07)	0 (0)	6 (16.66)
Professional training	13 (41.93)	7 (13.46)	3 (21.43)	12 (33.33)
University studies	12 (38.7)	3 (5.76)	0 (0)	2 (5.55)
Employment situation				
Student	0 (0)	3 (5.77)	1 (7.14)	0 (0)
Active	7 (22.64)	25 (48.08)	4 (28.57)	23 (63.88)
Unemployed	18 (58.01)	23 (44.23)	8 (57.15)	11 (30.56)
Retired	6 (19.35)	1 (1.92)	1 (7.14)	2 (5.56)
Noise exposure				
No	28 (90.33)	28 (53.85)	13 (92.86)	18 (50)
Yes, only in the past	0 (0)	15 (28.85)	1 (7.14)	8 (22.22)
Yes, currently	3 (9.67)	9 (17.30)	0 (0)	10 (27.78)
Use of hearing protectors				
No	29 (93.55)	45 (86.54)	10 (71.43)	31 (86.11)
Yes, partially	2 (6.45)	5 (9.62)	4 (28.57)	5 (13.89)
Yes, always	0 (0)	2 (3.84)	0 (0)	0 (0)
F.H. of depression	2 (6.45)	9 (17.3)	1 (7.14)	4 (11.11)
F.H. of early deafness	1 (3.22)	10 (19.23)	1 (7.14)	4 (11.11)
Right laterality	31 (100)	46 (88.46)	14 (100)	34 (94.44)
P.H. of significant pain				
No	18 (58.07)	26 (50)	9 (64.29)	19 (52.77)
Yes, only in the past	7 (22.58)	6 (11.54)	5 (35,71)	14 (38.88)
Yes, currently	6 (19.35)	20 (38.46)	0 (0)	3 (8.33)
P.H. of immunological alteration				
No	18 (58.07)	35 (67.30)	9 (64.29)	25 (69.44)
Yes, only in the past	7 (22.58)	5 (9.62)	0 (0)	4 (11.11)
Yes, currently	6 (19.35)	12 (23.08)	5 (35.71)	7 (19.45)
P.H. of significant ENT pathology				
No	31 (100)	49 (94.23)	14 (100)	33 (91.67)
Yes, only in the past	0 (0)	3 (5.77)	0 (0)	3 (8.33)
P.H. of mental pathology				
No	11 (35.48)	26 (50)	8 (57.14)	17 (47.22)
Yes, only in the past	20 (64.52)	20 (38.46)	6 (42.86)	17 (47.22)
Yes, currently	0 (0)	6 (11.53)	0 (0)	2 (5.56)
Analgesics treatment				
No	19 (61.29)	36 (69.23)	13 (92.86)	31 (86.11)
Yes, only in the past	0 (0)	2 (3.85)	0 (0)	2 (5.55)
Yes, currently	12 (38.71)	14 (26.92)	1 (7.14)	3 (8.34)
Immune treatment				
No	31 (100)	49 (94.23)	14 (100)	33 (91.67)
Yes, only in the past	0 (0)	0 (0)	0 (0)	1 (2.77)
Yes, currently	0 (0)	3 (5.77)	0 (0)	2 (5.55)
Anxiolytics treatment				
No	18 (58.07)	37 (71.15)	12 (85.71)	32 (88.88)
Yes, only in the past	3 (9.68)	3 (5.77)	0 (0)	2 (5.56)
Yes, currently	10 (32.25)	12 (23.08)	2 (14.29)	2 (5.56)
Antidepressants treatment				
No	21 (67.74)	37 (71.15)	14 (100)	30 (83.32)
Yes, only in the past	3 (9.68)	5 (9.62)	0 (0)	3 (8.34)
Yes, currently	7 (22.58)	10 (19.23)	0 (0)	3 (8.34)
Psychological treatment				
No	25 (80.65)	35 (67.31)	9 (64.29)	30 (83.33)
Yes, only in the past	6 (19.35)	11 (21.15)	5 (35.71)	6 (16.66)
Yes, currently	0 (0)	6 (11.54)	0 (0)	0 (0)
Taste in music				
No	0 (0)	10 (19.23)	0 (0)	4 (11.11)
Yes, listening to it regularly	24 (77.42)	30 (57.69)	10 (71.43)	22 (61.11)
Yes, but not listening to it regularly	7 (22.58)	12 (23.08)	4 (28.57)	10 (27.78)

Age is expressed as a mean (standard deviation). The rest of the variables are expressed as sample size: *n* (%). Interv.: Intervention.; F.H.: Family history; P.H.: Personal history; ENT: Ear Nose Throat.

**Table 2:** Responses to the auditory intervention of the different groups.

Groups	TOTAL Response to:	PARTIAL Response to:
Emotional Well-being Group	HAS-Psychological at 3 months HAMD at 6 months	
Anxiety and/or Depression Group	HAS-Total at 3 months HAS-Psychological at 3 months HAS-Somatic at 3 months	HAS-Total at 6 months HAS-Somatic at 6 months HAMD at 3 months HAMD at 6 months

Total Response: decrease ≥50% of the initial scale score; Partial Response: decrease between 25–49% of the initial scale score. The results are those that present a statistically significant difference between having been exposed to the auditory intervention and those who were not (*p* < 0.05).

**Table 3:** Standardized means differences of the different groups.

	T3m		T6m	
	HAS-Total	HAMD	HAS-Total	HAMD
Emotional Well-being groups with and without auditory intervention.	−1.16	−0.74	−0.68	−0.51
Anxiety and/or Depression groups with and without auditory intervention.	−0.20	−0.09	−0.01	−0.02

Cohen’s D, calculated with combined standard deviation for groups of different sizes. T3m: three months after the start; T6m: six months after the start; HAS: Hamilton Anxiety Scale; HAMD: Hamilton Depression Rating Scale.

**Table 4:** Demographic and clinical factors that predict a better or worse evolution of anxiety and depression symptoms over time, with and without auditory intervention.

	After 3 Months	After 6 Months
HAS: Psychol. Anxiety		
(Ω)	No findings	No findings
HAS: Somatic Anxiety		
(Ω)	No findings	No findings
HAS: Total Anxiety (*)		
Group without Intervention		
Better results	Having university studies compared to the rest of studies variables.	
Worse results	Range 51–65 years old. Being retired. History of pain. History of mental pathology. Having taken antidepressants in the past. Having taken analgesics in the past. Having followed psychological treatment in the past. Being retired.	Family history of depression.
Group with Auditory Intervention		
Better results	Being unemployed is better than working for someone else.	Age range 31–50 years old better than 51–65 years old.
Worse results	Having taken anti-anxiety or antidepressants in the past.	Having basic studies is worse than the rest of studies variables. Having taken anti-anxiety or antidepressants in the past.
HAMD (*)		
Group without Intervention		
Better results		
Worse results	History of chronic pain. Having taken analgesics in the past. Having followed psychological treatment in the past. Being retired.	
Group with Auditory Intervention		
Better results		Being unemployed is better than working for someone else.
Worse results		The current history of mental pathology presents a worse outcome than having had it in the past or not having had it at all.

Two-way ANOVA with repetitive measures in a single factor and Bonferroni post-test, (*) *p* < 0.05. (Ω) *p* > 0.05. HAS: Hamilton Anxiety Scale; HAMD: Hamilton Depression Rating Scale; Psychol.: Psychological.

## Data Availability

Data are available in the Supplementary Materials.

## Supplementary Materials

The following supporting information can be downloaded at: https://www.mdpi.com/article/10.3390/jcm14010288/s1, Document S1: Song list (modified Bérard Method); Document S2: Original. Data are available as attachments.
