# Supplementary material for: Approach to Mental Health Through a Frequency Modulated Auditory Intervention: A Controlled and Randomized Clinical Trial
Source: J Clin Med. 2025 Jan 6;14(1):288. doi: 10.3390/jcm14010288 (PMC11721829; doi:10.3390/jcm14010288)
Supplement: Supplementary file 1 [file jcm-14-00288-s001.zip › jcm-3386423-supplementary/S1. Song list (modified Bérard Method).pdf]

Tracklisting

Day 1: ……………………………………………………………………………….

- Session 1: Strauss J. Valses………………………………………………...
- Session 2: Brahms J. Symphony nº 1…………………………………………….

Day 2: ………………………………………………………………………………...

- Session 3: Zarzuela. Zarzuela Preludes …………………………………….
- Session 4: Chaikovski P.I. Nutcracker and Swan Lake …………………

Day 3: ……………………………………………………………………………….

- Session 5: Bruce Springsteen. Ocean surf…………………………………….
- Session 6: Schubert. Symphony nº 9……………………………………………...

Day 4: ………………………………………………………………………………...

- Session 7: Rimsky-Korsakov. Sherezade………………………………………
- Session 8: Natalie Cole. Greatest Hits…………………………………………

Day 5: ……………………………………………………………………………….

- Session 9: Liszt F. Prometeo – Tasso………………………………………….
- Session 10: Falla M. El amor brujo – El sombrero de tres picos……………..
